# Landscape genetics identified conservation priority areas for blue sheep (*Pseudois nayaur*) in the Indian Trans-Himalayan Region

**DOI:** 10.1038/s41598-023-44823-y

**Published:** 2023-10-24

**Authors:** Stanzin Dolker, Gul Jabin, Sujeet Kumar Singh, Bheem Dutt Joshi, Vinaya Kumar Singh, Supriyo Dalui, Kailash Chandra, Lalit Kumar Sharma, Mukesh Thakur

**Affiliations:** 1 Zoological Survey of India, New Alipore, Kolkata, 700053 West Bengal India; 2 Department of Zoology, University of Calcutta, Kolkata, 700019 West Bengal India; 3 Amity Institute of Forestry and Wildlife, Amity University Campus, Sector-125, Noida, 201303 UP India

**Keywords:** Conservation biology, Ecological modelling, Population genetics

## Abstract

The trans-Himalayan region of India, although have xeric features, still supports a unique assemblage of biodiversity, including some of the charismatic and endemic species. In the present study, we studied blue sheep (*Pseudois nayaur*) across the distribution range in the Western trans Himalayas of India and found about 18,775 km^2^ area suitable for blue sheep. The explicit Bayesian based spatial and non-spatial population structure analysis assigned blue sheep into two genetic populations, i.e., Ladakh and Lahaul-Spiti. We found relatively high genetic divergence in blue sheep which is also supported by the low current flow in Circuitscape model. With the multiple evidences, we explain landscape resistance facilitated by the landscape heterogeneity, and large patches of unsuitable habitats forced population divergence and poor functional connectivity. We found that blue sheep population has been demographically stable in the past, but showed a slight decline within the last few decades. This study is the first range-wide attempt to exhibit landscape features in shaping the spatial distribution, genetic structure and demography patterns of blue sheep in Western Himalayas, and will be of use in the conservation and management planning of blue sheep.

## Introduction

Heterogeneity in large landscape often delineate species boundary and primarily forms the foundation of genetic differentiation in free ranging wildlife. In this context, human-induced habitat loss and climate change are known to be the key threats to biodiversity conservation worldwide^1,2^. Despite the profound impact of such changes on global biodiversity, there is a scarcity of direct evidence on how species respond to such changes, while landscape changes (also due to climate change) are more evident and visible than the species adaptive response. Therefore, to facilitate long term persistence of a species, functional connectivity need to ensured so as to enable gene flow and sharing of alleles throughout the landscape^3,4^. In free ranging terrestrial wildlife, gene flow is often limited by the degree of landscape permeability and connectivity between different habitats patches^5^. Assessing the factors impacting habitat connectivity and dispersal are essential measure to interpret evolutionary consequences and to forbid local and regional extirpations. Landscape genetics is a trans-disciplinary approach, particularly useful in elucidating the cumulative influence of ecological, landscape and other anthropogenic factors on gene flow and natural selection^6–8^. In this context, maternally inherit mtDNA and nuclear microsatellite markers are used in addressing phylogeography, evolutionary history and current population genetics parameters with respect to the landscape features and habitat connectivity^6,9^. With the ensemble approach of coupling environmental, ecological and other anthropogenic variables with the genetic information of the natural populations may depict the current spatial genetic structure and provide empirical information of management interventions e.g., assigning large geographical barriers^10^, finding unique taxonomic units^11^ and identifying habitats for population^6–8^.

Blue sheep (*Pseudois nayaur)* is one of the widely distributed medium-sized alpine ungulate distributed from the Qilian Mountains in the north to The Himalayas in the south. Globally, it is distributed in China, Bhutan, India, Myanmar, Nepal, Pakistan and Tajikistan^12,13^. In India, it is distributed in Ladakh, Himachal Pradesh, Uttarakhand, Sikkim and Arunachal Pradesh between the elevation ranges from 2500 to 5500 m asl^14–16^. They typically prefer plateau, alpine barren and inter-valley grassland^16^ and play a key ecological role in the maintaining the mountain ecosystems by imparting a crucial prey base to the large carnivores and determine their density and distribution in that region^14,17,18^. Blue sheep are listed as least concern (LC) under the IUCN red list and listed as Schedule I under the Wildlife (Protection) Act, 1972 of India^16,19^. However, no firm information is available of the population size of blue sheep^19^. Previous studies reported climate change and urbanization are the major threats to blue sheep across distribution range and interestingly, blue sheep show relatively less seasonal altitudinal migration with respect to changing seasons ^20,21^. Moreover, blue sheep usually found in large herd and avoid steep slope. While, the mountain ranges of the Western Trans Himalayas of India i.e. the Ladakh and Zanskar range appear to separate the mountainous landscape by challenging topography such as steep cliffs and canyons that could act as barriers to blue sheep movement. In addition, blue sheep also compete with other mountain ungulates and livestock for limited resources and often impacted with their high densities^22–24^. In the present study, we adopted a landscape genetics approach to testify the functionality of biological corridor to support the movement and gene flow of blue sheep in Western trans-Himalaya.

## Results

**Figure 1:**
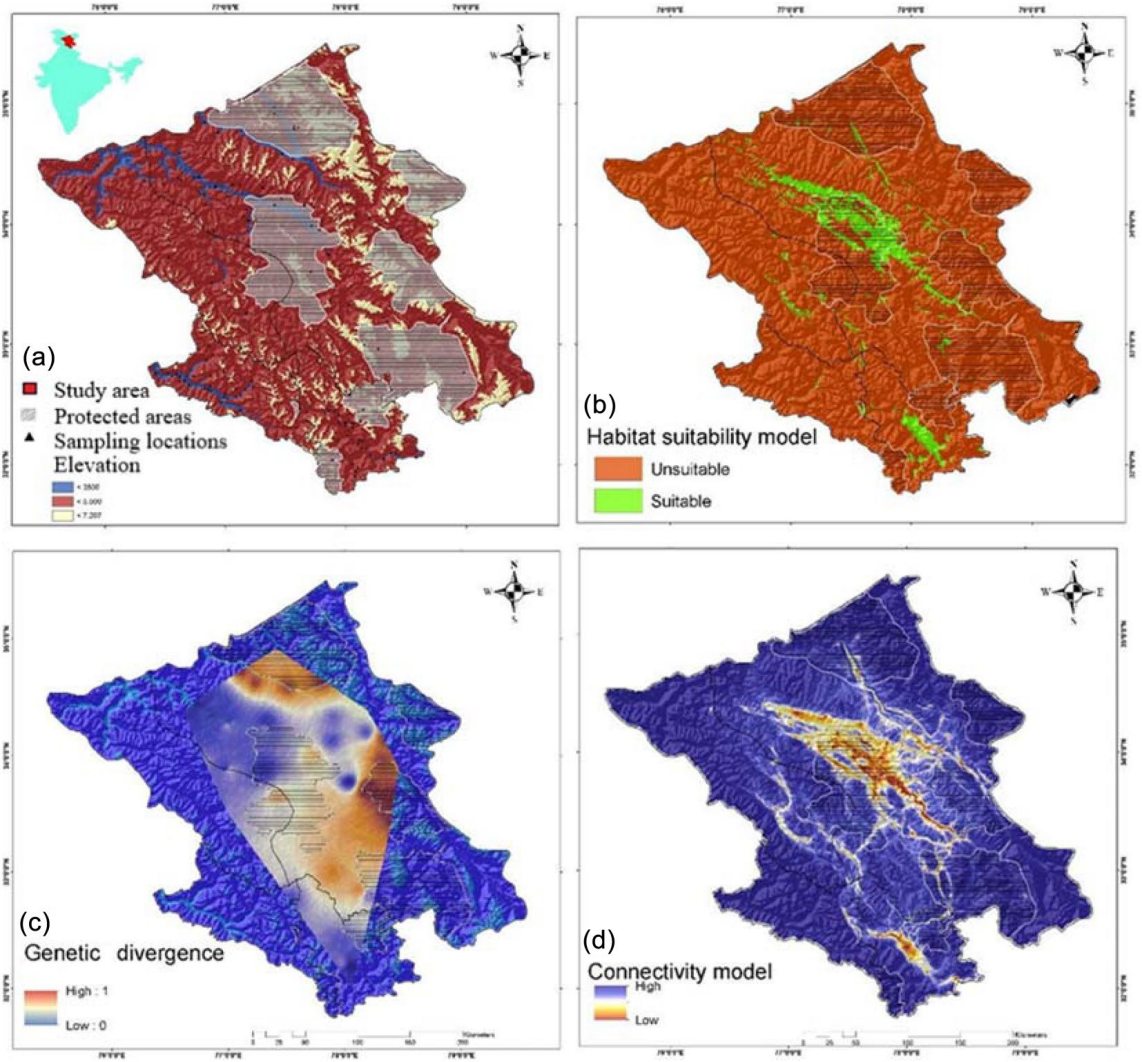
(**a**) Geographical map of the study area displaying sampling location of blue sheep (*Pseudois nayaur*) was created using ArcGIS 10.6 (http://www.esri.com) from Indian Trans-Himalayas. (**b**) Map of predicted habitat suitability for blue sheep in Ladakh and Lahaul-Spiti. The species distribution model of blue sheep was generated using Maximum Entropy species distribution model (MaxEnt v.3.3 http://biodiversityinformatics.amnh.org/open_source/maxent/) and ArcGIS 10.6 (http://www.esri.com) was used to visualize the map. (**c**) Genetic divergence plot of blue sheep in the studied landscape using the Genetic Landscape GIS Toolbox (https://www.sciencebase.gov/catalog/item/57e97d09e4b09082500c9210) in ArcGIS 10.6 (http://www.esri.com). High divergence at the peripheral population. (**d**) Map showing predicted corridors in the study landscape (High-brown and Low-blue). The connectivity map was generated with Circuitscape software v. 4.0 (https://circuitscape.org/downloads/) and plotted onto the map using ArcGIS 10.6 (http://www.esri.com).

Of about the 106,014 km^2^ of the study area, only 18,775 km^2^ (<20%) was found suitable for the blue sheep that lies primarily in Leh region of Ladakh (LA) and Spiti region of Lahaul-spiti (LS) (Fig. 1b). The estimated AUC was 0.89 ± 0.081 indicating all the selected variables significantly contributed to building the model. Jackknife estimates that most contributing variables was Precipitation Seasonality (16%) followed by Mean Temperature of Driest Quarter (13.2%) and Euclidean distance from grasslands (11.9%) while Topography influence index and Euclidean distance from barren land showed least contribution 1.3% and 1.1% respectively (Table S4, Fig. S1).

**Figure 2:**
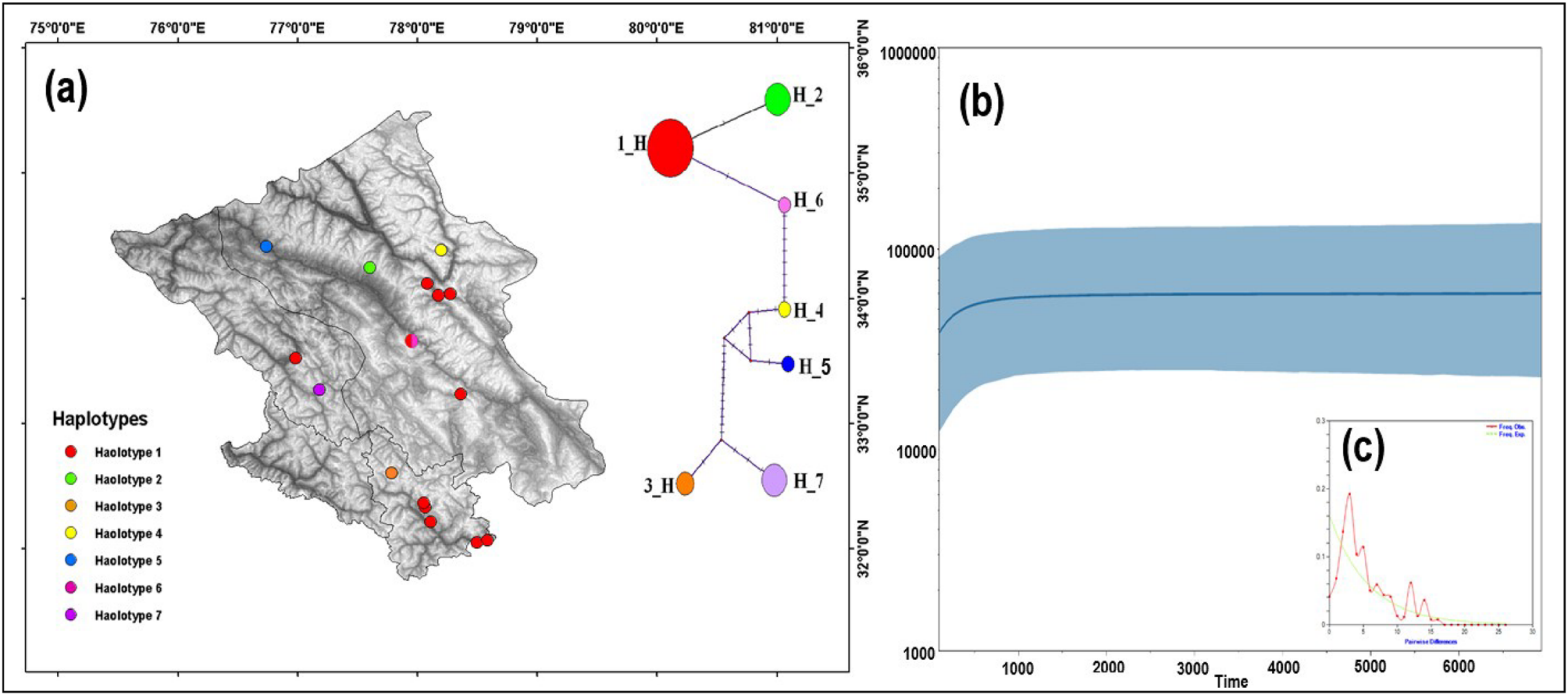
(**a**) Haplotype network generated in NETWORK software (v 10.2) using cytb gene of blue sheep in Ladakh and Lahaul-Spiti regions. The generated haplotype was then visualized on the map to understand the sharing of the haplotype in the studied area using ArcGIS 10.6 (ESRI, Redlands, CA). (**b**) Demographic history of *(Pseudois nayaur)* population estimated using Bayesian skyline plot in BEAST software v 2.6.3 https://www.beast2.org/ (Bayesian skyline plot showing a stable population in the recent past). The solid line is the median estimates of Ne τ (Ne = effective population size; τ = generation time), and the grey lines around median estimates is the 95% highest posterior density (HPD) estimate of the historic effective population size. The timing of events was estimated assuming a substitution rate of 2.0 × 10^–6^ substitutions/site/year (Joshi et al. 2020; Johns et al.1998). (**c**) The multimodal pattern of mismatch distribution was generated using DnaSP (v 6.12.03 http://www.ub.edu/dnasp/), indicating the blue sheep population under demographic equilibrium.

On analysing 137 sequences of cytochrome b gene of blue sheep, we obtained seven haplotypes (Fig. 2a) with 0.331 ± 0.095 and π 0.012 ± 0.00393, respectively (Table 1). No statistical significance for Tajima’s D and Fu and Li’s F and D test were detected (> 0.01). We did not find blue sheep in Kargil and Lahaul region as none of the faecal samples was identified as blue sheep. The studied population showed a multimodal pattern of mismatch distribution (Fig. 2c). However, the BSP indicated a demographically stable population with slight decline during the last few decades (Fig. 2b).

**Table 1:** Summary of molecular genetic diversity and neutrality tests of demography of blue sheep (*Pseudois nayaur*).

Diversity indices						Neutrality tests		
N	P	H	K	Hd	π	Tajima's D	Fu and Li's F	Fu and Li's D
137	22	7	4.227	0.331 ± 0.095	0.012 ± 0.00393	− 0.650*	1.002*	1.688*

*N* number of samples, *P* polymorphic sites, *H* number of haplotypes, *K* average number of nucleotide differences, *Hd* haplotype diversity, *π* nucleotide diversity.

P > 0.10 (*non significant).

We generated 137 consensus genotypes with nine loci with considerably good amplification success (≥ 80%) (Table 2). The ADO and FA in all the loci were non-significant. Majority loci (8/9) were highly informative (PIC value > 0.5) and all the loci deviated from HWE except BM1824 (P < 0.05). None of the pairs of loci showed significant linkage disequilibrium (P < 0.05).

**Table 2:** Genetic polymorphism of the blue sheep (*Pseudois nayaur*) population at nine microsatellite loci.

Locus	Na	Ne	Ho	He	uHe	PIC	Fis	PID (locus)	PID (sibs) (locus)	PID (cum)	PID (sibs) (cum)	FA	ADO
INRA35*^¶^	20	5.965	0.237	0.832	0.837	0.844	0.722	4.20E−02	3.40E−01	4.20E−02	3.40E−01	0.1	0.08
ETH152*^¶^	16	7.082	0.366	0.859	0.863	0.841	0.554	3.50E−02	3.30E−01	1.50E−03	1.10E−01	0.3	0.07
CSSM19*^¶^	15	5.708	0.623	0.825	0.829	0.804	0.195	4.90E−02	3.50E−01	7.30E−05	4.00E−02	0.09	0
CSRP6*^¶^	14	5.65	0.519	0.823	0.827	0.803	0.33	5.00E−02	3.50E−01	3.60E−06	1.40E−02	0.1	0
CSSM14*^¶^	13	4.372	0.658	0.771	0.775	0.735	0.105	8.30E−02	3.90E−01	3.00E−07	5.40E−03	0.2	0.07
ETH225*^¶^	13	3.973	0.243	0.748	0.752	0.729	0.658	8.90E−02	4.00E−01	2.70E−08	2.10E−03	0.03	0
ETH10*	11	3.943	0.268	0.746	0.75	0.703	0.617	1.10E−01	4.00E−01	2.90E−09	8.60E−04	0	0
Haut14*	15	3.4	0.317	0.706	0.709	0.671	0.534	1.10E−01	4.20E−01	3.00E−10	3.70E−04	0.38	0.07
BM1824	6	1.991	0.443	0.498	0.5	0.465	0.099	3.00E−01	5.80E−01	9.00E−11	2.10E−04	0.02	0
Mean	13.66	4.676	0.408	0.757	0.76		0.424						
SE	1.269	0.519	0.054	0.036	0.037		0.081						

*Na* observed number of alleles, *Ne* effective number of alleles, *Ho* observed heterozygosity, *He* expected heterozygosity, *PIC* polymorphic information content, *Fis* inbreeding coefficient index, *P*_*ID*_* (locus)* probability of identity (locus), *P*_*ID*_* sibs (locus)* probability of identity for sibs (locus), *P*_*ID*_* (cum)* probability of identity (cumulative), *P*_*ID*_* sibs*^*10*^*(cum)* probability of identity for sibs (cumulative).

^¶^Locus used for individual identification.

*HWE deviation (P value < 0.5).

Unique individuals were identified using a panel of six microsatellite loci and the cumulative probability of identity assuming all individuals were siblings (P_ID_ sibs) was 2.1 × 10^–3^ (2.1 match in 1000 genotypes). In total, 118 unique individuals were identified out of 137 consensus genotypes generated. Genetic diversity indices of blue sheep population showed a mean Na and Ne of 9.89 ± 1.15 (LA) to 10 ± 0.71 (LS) and 4.37 ± 0.58 (LA) to 3.80 ± 0.50 (LS), respectively. The mean Ho was 0.44 ± 0.06 in LA and 0.35 ± 0.06 in Spiti, while the mean He was 0.72 ± 0.05 in LA and 0.70 ± 0.03 in Spiti (Table S6). A broad variation inbreeding coefficient value among loci ranging from 0.099 (BM1824 locus) to 0.722 (INRA35 locus) indicates slight inbreeding between the populations (Table 2).

STRUCTURE analysis identified two genetic clusters (ΔK = 2). The genetic partitioning showed the presence of geo-spatial structure in the population i.e. 56 individuals from LA assigned to Cluster I while 47 individuals from LS were assigned to Cluster II (Fig. 4a). Only 14 (#8 individuals from LA and #6 individuals from LS) showed admixed ancestry. GENELAND inferred the presence of four genetic clusters based on the highest average posterior probability where majority of individuals were assigned into two clusters supporting the geo-spatial clusters i.e., LA and LS. The LA population showed a further break-down into four smaller groups, indicating the presence of meta-populations of blue sheep in LA (Fig. 4c). The multi variation DAPC also show similar clustering pattern to STRUCTURE and identified two major clusters (Fig. 4b).

**Figure 3:**
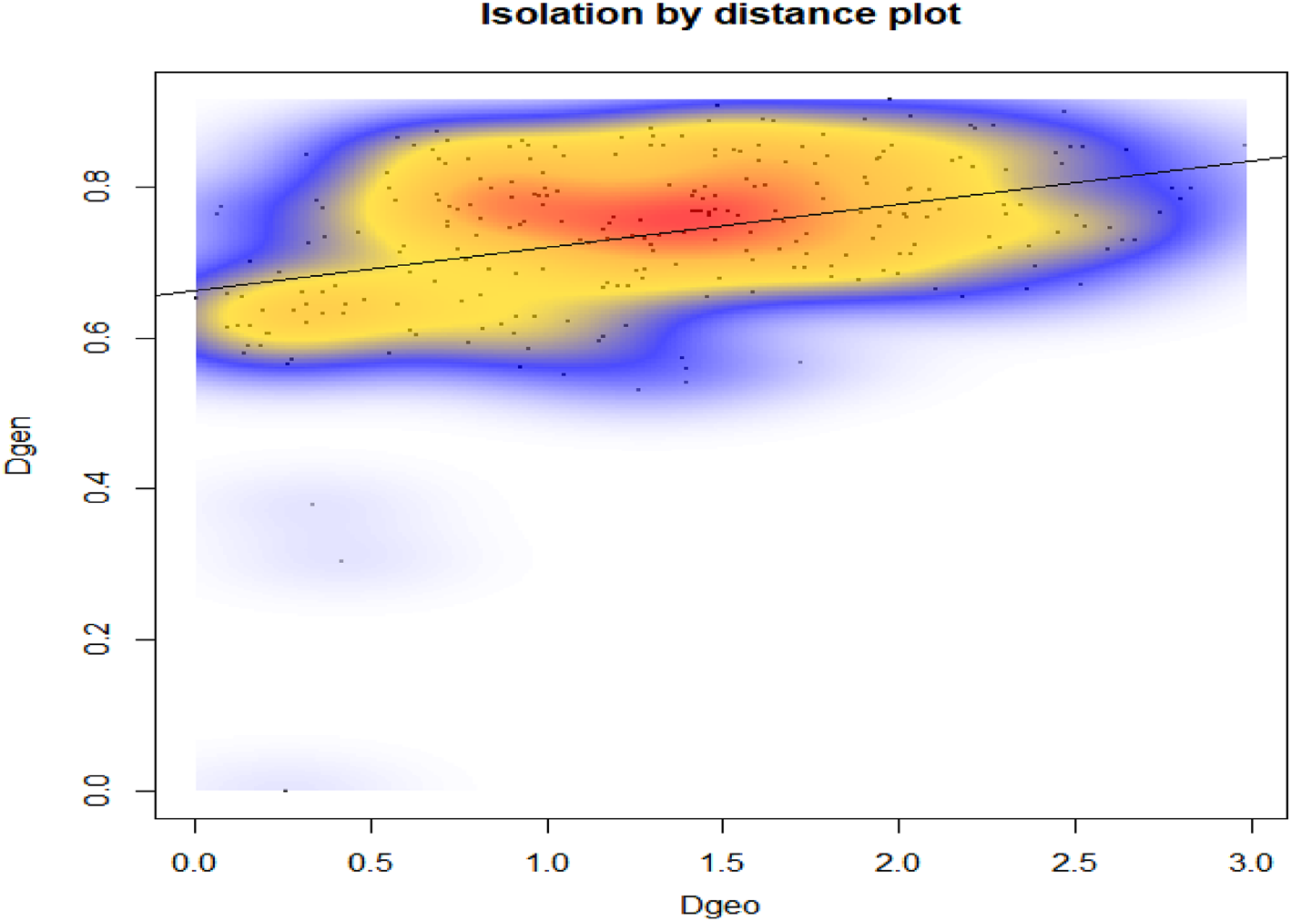
Scatterplot showing the result of Mantel test for presence of IBD (Isolation by distance) between significance of geographical distance on the genetic distance (r = 0.291) in the blue sheep population. Colour represent the relative density of point higher densities are represented by warmer colour while show the correlation between the distance matrices. IBD was performed in R.4.0 software with Adegenet version 1.3.4 package.

**Figure 4:**
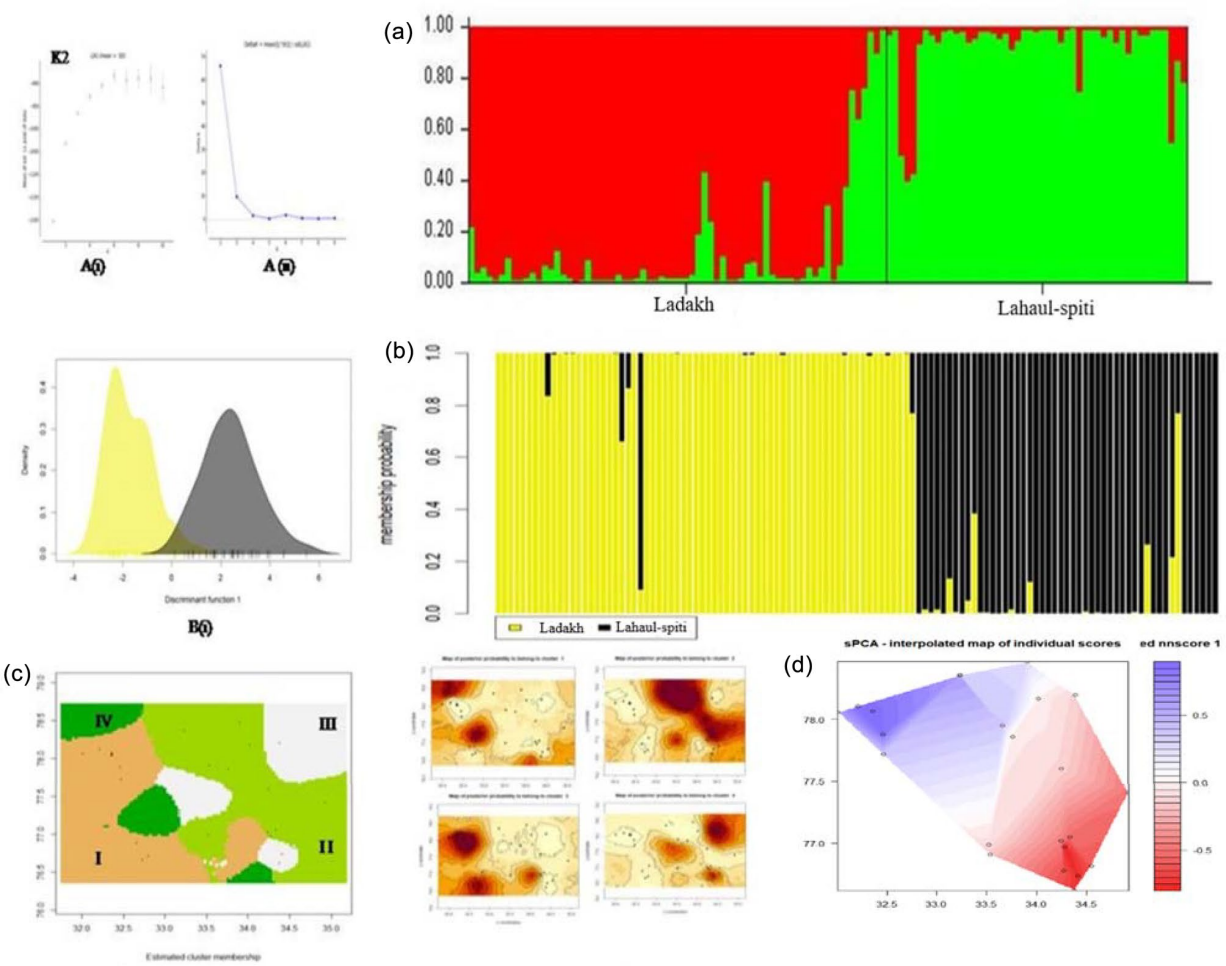
(**a**) Bayesian clustering patterns of blue sheep population at K2; Assignment at population level (cluster 1: Ladakh, cluster 2: Lahaul-spiti); A (i): Mean L (K), A (ii) The ad hoc quantity (delta K) over 20 runs for each K value. The structure analysis was performed using STRUCTURE v 2.3.4. (**b**) Non-Bayesian pattern of blue sheep with DAPC, (**c**) Estimated cluster membership shows spatial distribution of the four inferred genetic clusters across the study landscape using 118 blue sheep individuals (K4) based on clustering Bayesian model GENELAND (GENELAND v 4.0.3 4 package of R https://www.sciencebase.gov/catalog/item/57e97d09e4b09082500c9210). (**d**) Interpolation using a globally weighted regression of component 1 score for sPCA and contours are component score representing similarity across the landscape. DAPC and sPCA generated with Adegenet version 1.3.4 package of R (https://CRAN.R-project.org/package=adegenet).

BAYESASS showed low migrations i.e. ≤ 0.1% from either side from LA to LS and vice versa. The sPCA revealed a cline in allele frequencies from LA to LS (Fig. 4d). While the mantel test detected a gradual pattern of IBD across the study (r = 0.291; P > 0.01) (Fig. 3). The genetic divergence model also corroborated with the IBD and showed high genetic divergence between the LA and LS region and very high divergence in the Nubra and Changtang valley of LA which could be due to the peripheral population and edge effect (Fig. 1c).

The Circuitscape model showed cumulative currents confining within LA and LS and no high intensity current flows between regions indicating the lack of physical connectivity between the regions (Fig. 1d).

## Discussion

Previous studies on blue sheep in India mainly focused on the ecological aspect and no study was conducted on the population genetics of blue sheep^15,22,25,26^. For accurate assessment of the spatial genetic patterns of blue sheep inhabiting Ladakh and Lahaul-spiti, we identified the landscape features that influenced genetic structure. Here, we included habitat and corridor modelling in addition to genetic data to understand the impact of landscape on the genetic diversity of blue sheep. In general, blue sheep avoids forested habitats, prefers alpine pasture and frequently found near the escape covers^16,17^. We also observed the similar patterns in the present study, as habitat suitability is positively influenced by the grassland area. Since, there is low human habitation in the studied landscape, the anthropogenic variables had insignificant effects on the blue sheep habitat, rather the habitat suitability was mainly influenced by the bioclimatic variable and land use (precipitation, temperature and grassland). Blue sheep prefers low precipitation and rely on the mean Temperature of Driest Quarter. Blue sheep are usually found in large herds and have relatively less dispersal which is also supported by our study showing low migration rate between the two regions i.e., Ladakh and Lahaul-Spiti (Home range: − 3.7 km^2^ male and females 2.9 km^2^ Cui^27^; Zhang et al.^28^). The present study suggested that low connectivity between LA and LS in addition to complex topography and high expanses among habitat patches.

We found comparable genetic diversity of blue sheep with previous studies^29^ and other caprinae species e.g., wild goats^30^; Hangul^31^; and Iberian ibex^32^. The neutrality tests were non-significant, indicating a stable population size. Mismatch distribution curve also revealed a stable population of blue sheep with no bottleneck in the past. The BSP analysis showed a stable population in the past with experiencing a slight demographic decline in the recent past. Thus, all the demographic data corroborate with each other. Mitochondrial data suggested gene flow in the past as one haplotype (hap_1) was distributed in all the region and Zanskar haplotype (hap_7) (Fig. 2a) is closer to the Spiti haplotype rather than the LA as geographically it is closer to Spiti.

Population genetic structure analysis based on microsatellite marker revealed all the individuals grouped into two distinct clusters with limited gene flow. One of the factors contributing to the limited gene flow between LA and LS area is likely to be limited physical corridor and patchiness in suitable area. The previous studies also suggested influence of geographic distance and landscape barrier on the genetic distance in blue sheep population^33^. Likewise, we also obtained a significant IBD and sPCA results suggested that individuals sampled on the periphery have higher genetic divergence than the central populations (Figs. 3, 4D). In addition to the landscape barrier, mating strategies and breeding system of blue sheep may also influence the genetic diversity between the landscapes^34^. The Zanskar range that divides the two regions could be resistance that impedes connectivity however, this needs a further detailed investigation. In other studies, much attention has been focused on the rare and specialist species while widely distributed species are often neglected and considered safe without recognizing their peripheral population^31^.

Despite being important biodiversity hotspot of unique and endemic fauna, not much attention has been given in the context of genetic monitoring of large mammals in these landscapes. Further, there are six protected areas in these landscapes where local live in harmony with the indigenous wildlife. Both, Changthang (CWS) and Karakoram (KWS) sanctuaries of LA where blue sheep majorly reported have no definite boundary till now. Moreover, these regions had encountered drastic changes in the last few years including increased presence of defense forces and large influx of tourists leading to unplanned construction that have disturbed the harmonious association and affected the native wildlife. A few earlier studies have identified that anthropogenic changes often restrict gene flow among populations and exhibited the utility of landscape genetics in conservation and management planning in ungulates^35^. Likewise, with the blend of genetic data and landscape modeling, we provide pragmatic potential connecting corridors supporting the movement and gene flow in blue sheep in the studied landscape. Although, we tested the present landscape genetic model on blue sheep, we believe the validated connecting corridors could be useful in multi species conservation and management.

## Materials and methods

### Study area

We prioritized sampling of blue sheep in the reported distribution of the Lahul and Spiti (LS) districts of Himachal Pradesh and Leh and Kargil districts of Ladakh union territory (LA-UT), falling under the Western Trans Himalayan region covering a total area of 1,06,014 km^2^ (LA 86,983 km^2^ and LS 19,032 km^2^) (Fig. 1). The Lahaul valley possesses mix condition of Himalaya and trans-Himalaya while Spiti and Ladakh regions harbour low faunal diversity and the trans-Himalayan features. The study landscape is consisting of six protected areas i.e. Pin Valley National Park (PNP), Kibber Wildlife Sanctuary KBS, and Chandra Tal Wildlife Sanctuary (CWS) located in Spiti valley of Himachal Pradesh and Changthang Cold Desert Sanctuary (CWS), Karakoram Wildlife Sanctuary (KWS), Hemis National Park (HNP) located in LA UT.

### Species occurrence data

Field surveys were performed from 2018 to 2021 in the studied landscape. We opted well established contour transect in the studied landscape and in total, 350 occurrence points of blue sheep were recorded (275 pellet samples, 75 sign survey). In addition to the primary occurrence records, secondary data was extracted from the Global Biodiversity Information Facility (GBIF) database (www.gbif.org). For distribution modelling, combination of primary as well as secondary data were used. To reduce over fitting of the model spatially independent locations were used after rectifying the location points based on the home range of blue sheep^27,28^.

### Ethics declarations

This study was based on samples collected non-invasively where no animal was captured or harmed. Thus, ethical clearance was not needed.

### Sampling and DNA extraction

We collected 275 non-invasive (pellet) samples, i.e., 118 pellet samples from LS region and 157 samples from LA region possibly belonging to blue sheep (Fig. 1a). The collected samples were sun dried and stored in sterile containers with ¼ filled with silica crystals. Genomic DNA from pellets was extracted using QIAamp Fast DNA Stool Mini Kit (QIAGEN, Germany) following manufacturer's instructions.

### Species identification

For species verification, we used partial fragments of the mitochondrial cytochrome b^36^. We set-up the PCR in a 10μL containing 10X PCR buffer, 2 mM MgCl2, 2.5 mM dNTPs mix, 0.1 μM primers each, and 20-30 ng template. The amplification conditions were as follows: 94 °C for 5 min followed by 35 cycles at 94 °C for 30 s, 53 °C for 45 s, and 72 °C for 45 s, and a final extension at 72 °C for 10 min on a Veriti Thermocycler (Applied Biosystems, USA). The sanger sequencing was performed following Big-Dye Terminator Cycle Sequencing Kit version 3.1 (Thermo Scientific, USA) after exo-sap clean up on an ABI 3730 Genetic analyzer (Applied Biosystems, USA).

### Microsatellite genotyping

All the genetically identified samples of blue sheep were processed for genotyping. We selected 22 microsatellites (15 loci of Bovidae;^30^ and 7 loci of cervidae;^37^ (Table S1). Total, 16 microsatellites were successfully amplified (Table S2). PCRs were set up following Gao et al.^33^ (Table 2). The fluorescence-based genotyping was performed on Genetic Analyzer (Applied Biosystem, Foster City, USA) and allele scoring was performed using Gene Mapper (version 4.1, Applied Biosystems, USA).

### Data analysis

#### Habitat suitability model

To identify suitable habitat for blue sheep, we used a Maximum Entropy (MaxEnt) species distribution model (version 3.3^38^). We started with a set of 30 habitat variables including present bioclimatic variable, topographic variables, physiological variables and anthropogenic variables. The 19 bioclimatic variables were obtained from the WorldClim data base at 30 arc second scale (https://www.worldclim.org/)^39^ (Table S3). Topographic variables such as elevation, slope, aspect, ruggedness and topography wetness index were generated from digital elevation model using ArcGIS 10.6 software (ESRI, REDLANDS, CA). MODIS data were downloaded for land use/land cover (LULC) from USGS Earth Explorer (https://earthexplorer.usgs.gov) and ArcGIS 10.6 (ESRI, REDLANDS, CA) was used for calculating the Euclidian distance. Human influence index was downloaded from Socioeconomic Data and Application (SEDAC). The variables were resampled at 1 km resolution and converted to ASCII format in ArcGIS 10.6 (ESRI, REDLANDS, CA). Multi- collinearity among the variables were tested in the R platform using ENM tool^40^ and the variable with Person Correlation Coefficient (r) more than 0.8 were dropped from the analysis. Finally, eighteen spatially independent predictors (Table S4) were used for mapping the suitable habitat of blue sheep and best fit model was determined in the R package ENMeval^41^ on the basis of lowest Akaike information criterion (AICc) value^42^. Further, for the model evaluation, we used 70% of species presence points as training data and 30% as testing data. The modelling was assessed based on Receiver Operating Characteristics (ROC) Area under the curve (AUC) with the threshold value higher than 0.8 < AUC < 1 which provide good prediction model.

#### Sequencing data

Generated sequences were cleaned with Sequencher 5.4.6 (Gene Code Corp) and validated through the BLAST tool of NCBI/GenBank. The identified sequences were aligned by multiple sequence alignment using CLUSTAL W in MEGA version X^43^ and trimmed to generate similar length of sequences for further genetic analysis.

Using the program DnaSP version 6.12.03^44^, Neutrality tests (Tajima’s D, Fu and Li’s F and D statistics) and genetic diversity indices i.e. no. of haplotypes (H), nucleotide diversity (π), haplotype diversity (Hd), average number of nucleotide differences (K) and mismatch distribution test for demographic expansion, equilibrium or bottleneck were computed. The NETWORK^45^ software was used to build a median-joining network. Subsequently, the obtained haplotype was then plotted on the map to visualize the haplotype sharing across the studied areas, using ArcMap 10.6 (ESRI, REDLANDS, CA). Bayesian Skyline Plot (BSP) was constructed to determine the history of population over times using BEAST software version 2 6.3^46^ by Bayesian Skyline method^47^. The HKY model and the Markov Chains Monte Carlo (MCMC) were run for 10^8^ steps that yielded effective sample sizes (ESS) under a strict molecular clock and a stepwise skyline model. The molecular evolution was calibrated assuming a substitution rate of 2.0E−7 substitutions/ site/year following^11,48^ and the results were visualized using Tracer version 1.7^49^.

#### Microsatellite data

For individual identification, we selected a panel of six loci on the basis of their least or no genotyping error, relatively short amplicon size, high amplification success and a high discriminating power to avoid the overestimation of individuals. The locus wise and cumulative probability of identity for unrelated individuals (P_ID_) and siblings (P_ID_ sibs) were calculated in GenAlEx version 6.51^50^ and the number of unique individuals were estimated in GeneCap version 1.2.2^51^ and the PIC value for each locus was estimated using CERVUS version 3.0^52^. While the nine markers were used to calculate the genetic diversity indices that included numbers of observed (N_a_) and effective alleles (N_e_), observed heterozygosity (H_O_), expected heterozygosity (H_E_), and Wright's inbreeding coefficient at each locus utilizing GENAIEX version 6.5^50^. We tested the deviation of any loci from Hardy–Weinberg equilibrium (HWE) using the program FSTAT version 2.9.4^53^ and null allele frequencies were calculated using CERVUS version 3.0^52^. Linkage disequilibrium (LD) was tested using GENEPOP version 4.7 following 10,000 dememorizations, 100 batches and 10,000 iterations per batch^54^.

To determine whether discrete population structure was present in the blue sheep population, we used two Bayesian clustering (STRUCTURE and GENELAND) and one Non-Bayesian clustering (DAPC) method. First Bayesian clustering approach was performed in STRUCTURE version 2.3.4^55^ with a burn-in period of 50,000 and 500,000 (MCMC) repetitions and 20 independent replicates at each ‘K’. The appropriate K value was determined by calculating ad hoc quantity (ΔK)^56^. Individuals were assigned to the inferred clusters using a threshold proportion of membership (q) i.e., q ≥ 0.80, otherwise an individual was determined as admixed if the q value was less than 0.80^57–59^. Secondly to spatially explicit the genetic discontinuities between the populations, we performed Bayesian clustering analysis in GENELAND version 4.0.3^60^ through an extension of R package. In the analysis, along with multi-locus genotype, spatial coordinates were also taken into account. We conducted 20 independent runs for each K ranging from 1 to 10, with 1,000,000 iterations and 1000 thinning with other parameters were kept as default values (maximum rate of the Poisson process, 100; uncertainty on spatial coordinates, 0; maximum number of nuclei, 300; null allele model, FALSE). The runs showing the highest average logarithm of the posterior probability were selected and post-processing was conducted using 100 × 100 pixels in the spatial domain with a burn in period of 200. Finally non-Bayesian clustering methods i.e., DAPC were run to assign the possible clusters in Adegenet version 1.3.4 package of R^61^. To evaluate the migration rate between the LA and LS population, we run BAYESASS version 1.3^62^ at 9 × 10^6^ MCMC iterations, 10^6^ were discarded as burn-in and sampling frequency was done at every 2000 intervals. Runs were carried out at migration rate prior of 0.05, while other parameters were kept at default.

### Corridor connectivity using landscape genetics

To explore the potential landscape barriers and corridors effecting the gene flow and population divergence of blue sheep, we first estimated genetic divergence in GenAlEx version 6.51^50^. Then, we mapped the pattern of genetic distances (independent of any a priori model of landscape resistance) using the Genetic Landscape GIS Toolbox^63^ in ArcGIS 10.6 (ESRI, REDLANDS, CA). We used the Single Species module, to calculate surface of the pair-wise population genetic differentiation by generating a network. Midpoint of each edge of the network becomes associated with genetic diversity value. In the package, both spatial location points as well as relative genetic divergence values were used. In addition, presence of Isolation by distance (IBD) across the study landscape was also generated with Mantel test. The Mental test and sPCA was performed in R.4.0 software with Adegenet version 1.3.4 package^61^. Lastly, we built connectivity model based on circuit theory in the Circuitscape software version 4.0^64^ to test the connectivity between the habitat patches to understand the dispersal and gene flow following Dalui et al.^6^. For conductance modelling resistance raster was constructed with ensemble of genetic divergence and habitat suitable model using the ArcGIS 10.6 software (ESRI, REDLANDS, CA) extension Gnarly landscape utilities package Resistance and Habitat Calculator^65^.

### Supplementary Information

Supplementary Information.

## Data Availability

The sampling location of blue sheep used in this study is available on DRYAR (10.5061/dryad.t1g1jwt7z). The novel seven haplotypes generated are available on NCBI and their unique accession numbers are mentioned under supplementary table (Table S5).
