# Supplementary material for: Landscape genetics identified conservation priority areas for blue sheep (Pseudois nayaur) in the Indian Trans-Himalayan Region
Source: Sci Rep. 2023 Oct 24;13:18152. doi: 10.1038/s41598-023-44823-y (PMC10598060; doi:10.1038/s41598-023-44823-y)
Supplement: Supplementary file 1 — Supplementary Information. [file 41598_2023_44823_MOESM1_ESM.docx]

### Supplementary file for Landscape genetics identified conservation priority areas for blue sheep (*Pseudois nayaur*) in the Indian Trans-Himalayan Region

### Stanzin Dolker, Gul Jabin, Sujeet Kumar Singh, Bheem Dutt Joshi, Vinay Kumar Singh, Supriyo Dalui, Kailash Chandra, Lalit Kumar Sharma and Mukesh Thakur.

**Author information**

Authors and Affiliations

1. **Zoological Survey of India, New Alipore, Kolkata, West Bengal, 700053, India**

Stanzin Dolker, Gul Jabin, Sujeet Kumar Singh, Bheem Dutt Joshi, Vinay Kumar Singh, Supriyo Dalui, Kailash Chandra, Lalit Kumar Sharma and Mukesh Thakur**.**

1. **Department of Zoology, University of Calcutta, Kolkata, West Bengal, 700019, India.**

Stanzin Dolker, Gul Jabin, Supriyo Dalui

1. **Amity Institute of Forestry and Wildlife, Amity University Campus, Sector-125, Noida, UP, 201303, India.**

Sujeet Kumar Singh.

Figure S1 to S2

Table S1 to S5

[Table S 1 : List of all predictor variables 4](#_Toc124431615)

[Table S 2: Environmental variables used for construct habitat suitable modeland the estimates of relative contributions to the Maxent model. 5](#_Toc124431616)

[Table S 3: Twenty-two microsatellites marker and their characteristics selected for blue sheep (Pseudo isnayaur) 6](#_Toc124431617)

[Table S 4: Four multiplex panels and three singleplexes of fourteen microsatellite markers used for Blue sheep. 7](#_Toc124431618)

[Table S 5:Accession number of Blue sheep (Pseudois nayaur) sequences submitted to the NCBI 8](#_Toc124431619)

[Table S 6: Genetic polymorphism of Blue sheep (*Pseudois nayaur*) population at nine microsatellite loci with two populations 9](#_Toc124431620)

**
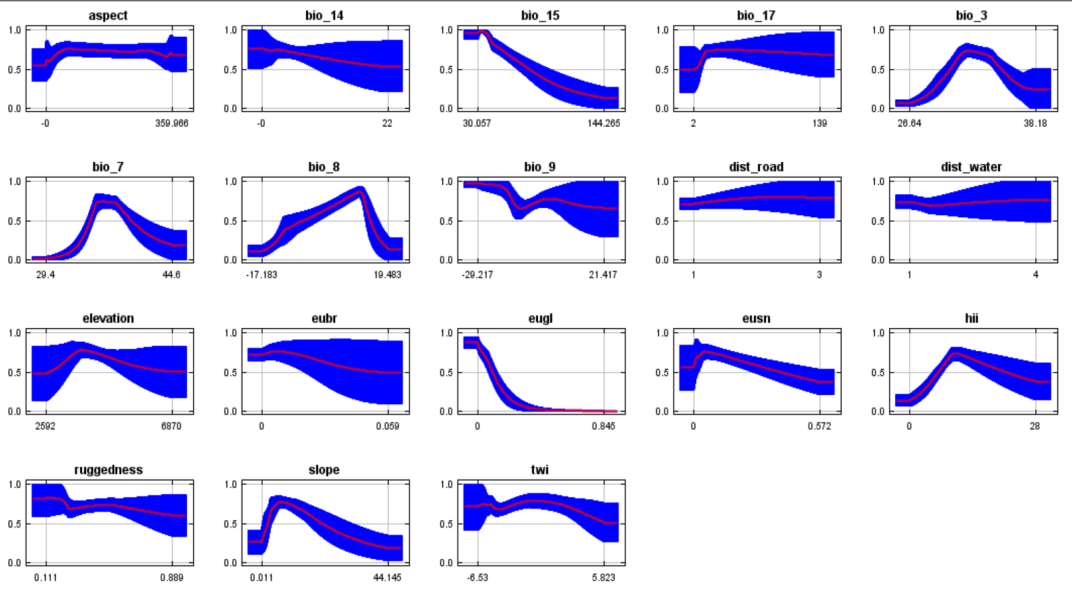
**

Figure S 1: Response curve showing how each environmental variable affects the MaXent prediction. The curves show the mean response of the 10 replicate MaXent runs (red) and the mean +/- one standard deviation (blue, two shades for categorical variables).

**
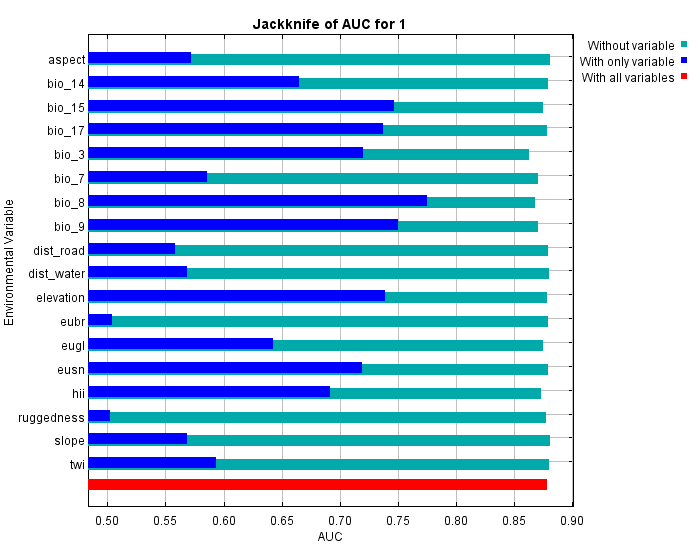
**

Figure S 2 : Jackknife test of variable importance, using AUC on test data.

Table S 1 : List of all predictor variables

| Variable | Code |
| --- | --- |
| Annual Mean Temperature | bio_1 |
| Mean Diurnal Range (Mean of monthly (max temp - min temp) | bio_2 |
| Isothermality (BIO2/BIO7) | bio_3 |
| Temperature Seasonality (Standard Deviation) | bio_4 |
| Max Temperature of Warmest Month | bio_5 |
| Min Temperature of Coldest Month | bio_6 |
| Temperature Annual Range (BIO5-BIO6) | bio_7 |
| Mean Temperature of Wettest Quarter | bio_8 |
| Mean Temperature of Driest Quarter | bio_9 |
| Mean Temperature of Warmest Quarter | bio_10 |
| Mean Temperature of Coldest Quarter | bio_11 |
| Annual Precipitation | bio_12 |
| Precipitation of Wettest Month | bio_13 |
| Precipitation of Driest Month | bio_14 |
| Precipitation Seasonality (Coefficient of Variation) | bio_15 |
| Precipitation of Wettest Quarter | bio_16 |
| Precipitation of Driest Quarter | bio_17 |
| Precipitation of Warmest Quarter | bio_18 |
| Precipitation of Coldest Quarter | bio_19 |
| Roughness | Ruggedness |
| Slope | slope |
| Hill shade | hills |
| Aspect | aspect |
| Elevation | Elev |
| Human influence index | HII |
| Topographic wetness index | TWI |
| Euclidean distance to forest | eufo |
| Euclidean distance to savanna | eusav |
| Euclidean distance to grass land | eugl |
| Euclidean distance to Barrens | eubr |
| Euclidean distance to bnow cover | eusn |
| Euclidean distance to road | dist_road |
| distance to water | dist_water |

Table S 2: Environmental variables used for construct habitat suitable model and the estimates of relative contributions to the Maxent model.

| **Variable** | **Percent contribution** | **Permutation importance** |
| --- | --- | --- |
| bio_15 | 24.5 | 16.5 |
| bio_3 | 16.9 | 11 |
| hii | 9.4 | 3.6 |
| bio_7 | 7 | 10.2 |
| bio_8 | 6.6 | 5.6 |
| eusn | 5.6 | 3.8 |
| eugl | 5 | 11.9 |
| slope | 4.2 | 3.4 |
| aspect | 3.7 | 3.4 |
| bio_9 | 3 | 13.2 |
| twi | 2.5 | 1.3 |
| bio_17 | 2.5 | 3.5 |
| dist_road | 2.1 | 2.3 |
| elevation | 1.6 | 3.1 |
| bio_14 | 1.4 | 4 |
| eubr | 1.3 | 1.1 |
| ruggedness | 1.3 | 0.6 |
| dist_water | 1.3 | 1.4 |

Table S 3: Twenty-two microsatellites marker and their characteristics selected for blue sheep (*Pseudois nayaur*)

| Microsatellite marker | Temperature (°c) | Expected range | References |
| --- | --- | --- | --- |
| Haut14 | 55 | 120-157 | Thieven et al. 1995 |
| INRA35 | 55 | 112-120 | Vaiman et al. 1994 |
| CSPS115 | 55 | 238-256 | Steffen et al. 1993 |
| CSSM19 | 53 | 150-160 | Moore et al. (1992,) |
| BM1818 | 55 | 249-272 | Bishop et al. 1994 |
| CSSM14 | 55 | 127-134 | Moore et al.  (1992, 1994), |
| HAUT 24 | 55 | 109-126 | Thieven et al. 1997 |
| TGLA53 | 55 | 133-152 | Georges and Massey 1992 |
| ETH10 | 57 | 205-220 | Solinas-Toldo et al. 1993 |
| INRA23 | 55 | 188-215 | Vaiman et al. 1994 |
| ETH225 | 53 | 127-145 | Steffen et al. 1993 |
| BM1824 | 55 | 160-171 | Bishop et al. 1994 |
| SPS115 | 55 | 239-245 | Moore etal. 1994 |
| SR-CSRP6 | 57 | 148-162 | Maudet et al., 2002 |
| ETH152 | 55 | 188-197 | Steffen et al. 1993 |
| BM203 | 55 | 218-231 | Bishop et al. 1994 |
| BM415 | 57 | 145-165 | Bishop et al. 1994 |
| BM2113 | NA | NA | Bishop et al. 1994 |
| TGLA126 | NA | NA | Georges and Massey 1992 |
| TGLA122 | NA | NA | Georges and Massey 1992 |
| ETH3 | NA | NA | Solinas-Toldo et al. 1993 |
| HEL1 | NA | NA | Kaukinen&Varvio 1993 |

Table S 4: Four multiplex panels and three singleplexes of fourteen microsatellite markers used for blue sheep (*Pseudois nayaur*).

| Multiplex (MP) | Loci ID | Dye color | Size range | Ta |
| --- | --- | --- | --- | --- |
| MP 1 | Haut14 | FAM | 120-157 | 55 |
|  | CSPS115 | FAM | 238-256 | 55 |
|  | BM1818 | NED | 150-160 | 55 |
|  | CSSM14 | VIC | 249-272 | 55 |
|  | INRA23 | VIC | 188-215 | 55 |
|  | BM1824 | NED | 160-171 | 55 |
| MP2 | INRA35 | FAM | 112-120 | 55 |
|  | SPS115 | NED | 239-245 | 55 |
|  | ETH152 | FAM | 188-197 | 55 |
| MP 3 | BM203F | VIC | 218-231 | 53 |
|  | CSSM19 | FAM | 150-160 | 53 |
|  | ETH225 | VIC | 127-145 | 53 |
| MP 4 | ETH10 | FAM | 205-220 | 57 |
|  | SR-CSRP6 | FAM | 239-245 | 57 |
| SP1 | HAUT 24 | FAM | 109-126 | 55 |
| SP2 | TGLA53 | FAM | 133-152 | 55 |
| SP3 | BM415 | FAM | 145-165 | 57 |

Table S 5:Accession number of blue sheep (Pseudois nayaur) sequences submitted to the NCBI

| **Haplotype no.** | **Accessions no.** |
| --- | --- |
| Hap-1 | OQ230775 |
| Hap-2 | OQ230776 |
| Hap-3 | OQ230777 |
| Hap-4 | OQ230778 |
| Hap-5 | OQ230779 |
| Hap-6 | OQ230780 |
| Hap-7 | OQ230781 |

Table S 6: Genetic polymorphism of Blue sheep (*Pseudois nayaur*) population at nine microsatellite loci with two populations

| POP | Locus | Na | Ne | Ho | He | uHe | PID ^(locus)^ | PID(sibs) (locus) | PID ^(cum)^ | PID(sibs) (cum) |
| --- | --- | --- | --- | --- | --- | --- | --- | --- | --- | --- |
| Ladakh | INRA35* | 17 | 4.32 | 0.279 | 0.769 | 0.775 | 7.70E-02 | 3.80E-01 | 7.70E-02 | 3.80E-01 |
|  | ETH152* | 10 | 6.11 | 0.344 | 0.836 | 0.843 | 4.60E-02 | 3.40E-01 | 3.60E-03 | 1.30E-01 |
|  | CSSM19* | 11 | 5.71 | 0.787 | 0.825 | 0.832 | 5.10E-02 | 3.50E-01 | 1.80E-04 | 4.60E-02 |
|  | CSRP6* | 10 | 6.53 | 0.565 | 0.847 | 0.854 | 4.20E-02 | 3.40E-01 | 7.60E-06 | 1.60E-02 |
|  | CSSM14* | 10 | 4.45 | 0.587 | 0.775 | 0.781 | 7.80E-02 | 3.80E-01 | 5.90E-07 | 6.00E-03 |
|  | ETH225* | 10 | 5.26 | 0.323 | 0.81 | 0.817 | 6.20E-02 | 3.60E-01 | 3.70E-08 | 2.10E-03 |
|  | ETH10* | 7 | 3.16 | 0.338 | 0.684 | 0.689 | 1.50E-01 | 4.50E-01 | 5.60E-09 | 9.60E-04 |
|  | Haut14* | 10 | 2.18 | 0.322 | 0.542 | 0.546 | 2.30E-01 | 5.40E-01 | 1.30E-09 | 5.10E-04 |
|  | BM1824 | 4 | 1.57 | 0.441 | 0.364 | 0.366 | 4.40E-01 | 6.80E-01 | 5.70E-10 | 3.50E-04 |
|  | Mean | 9.89 | 4.37 | 0.44 | 0.72 | 0.72 |  |  |  |  |
|  | SE | 1.15 | 0.58 | 0.06 | 0.05 | 0.05 |  |  |  |  |
| Lahaul-Spiti | INRA35* | 11 | 5.9 | 0.167 | 0.831 | 0.842 | 4.80E-02 | 3.50E-01 | 4.80E-02 | 3.50E-01 |
|  | ETH152* | 15 | 5.96 | 0.4 | 0.832 | 0.843 | 4.30E-02 | 3.40E-01 | 2.10E-03 | 1.20E-01 |
|  | CSSM19* | 12 | 2.86 | 0.4 | 0.65 | 0.658 | 1.40E-01 | 4.60E-01 | 2.90E-04 | 5.50E-02 |
|  | CSRP6* | 11 | 3.09 | 0.457 | 0.677 | 0.684 | 1.30E-01 | 4.40E-01 | 3.70E-05 | 2.40E-02 |
|  | CSSM14* | 9 | 3.55 | 0.75 | 0.718 | 0.726 | 1.30E-01 | 4.20E-01 | 4.70E-06 | 1.00E-02 |
|  | ETH225* | 11 | 2.23 | 0.143 | 0.551 | 0.557 | 2.20E-01 | 5.30E-01 | 1.00E-06 | 5.50E-03 |
|  | ETH10* | 9 | 2.59 | 0.159 | 0.613 | 0.62 | 1.90E-01 | 4.90E-01 | 2.00E-07 | 2.70E-03 |
|  | Haut14* | 11 | 5.38 | 0.31 | 0.814 | 0.824 | 5.80E-02 | 3.60E-01 | 1.20E-08 | 9.60E-04 |
|  | BM1824* | 5 | 2.65 | 0.447 | 0.622 | 0.629 | 2.00E-01 | 4.90E-01 | 2.40E-09 | 4.70E-04 |
|  | Mean | 10.4 | 3.8 | 0.359 | 0.701 | 0.709 |  |  |  |  |
|  | SE | 0.9 | 0.5 | 0.06 | 0.03 | 0.04 |  |  |  |  |
| Total | Mean | 10.17 | 4.08 | 0.4 | 0.71 | 0.72 |  |  |  |  |
|  | SE | 0.71 | 0.38 | 0.04 | 0.03 | 0.03 |  |  |  |  |
